# Provision of digital devices and internet connectivity to improve synchronous telemedicine access in the U.S.: a systematic scoping review

**DOI:** 10.3389/fdgth.2024.1408170

**Published:** 2024-07-29

**Authors:** Joshua Bell, Laura M. Gottlieb, Courtney R. Lyles, Oanh Kieu Nguyen, Sara L. Ackerman, Emilia H. De Marchis

**Affiliations:** ^1^ Department of Pediatrics, University of California, San Francisco, CA, United States; ^2^ Department of Family & Community Medicine, University of California, San Francisco, CA, United States; ^3^ Social Interventions Research and Evaluation Network (SIREN), Center for Health and Community, University of California, San Francisco, CA, United States; ^4^ Center for Healthcare Policy and Research, University of California, Sacramento, CA, United States; ^5^ Department of Public Health Sciences, University of California, Davis School of Medicine, Davis, CA, United States; ^6^ UCSF Center for Vulnerable Populations, University of California, San Francisco, CA, United States; ^7^ Division of Hospital Medicine at Zuckerberg San Francisco General Hospital, Department of Medicine, University of California, San Francisco, CA, United States; ^8^ Chan Zuckerberg Biohub, San Francisco, CA, United States; ^9^ Department of Social & Behavioral Sciences, School of Nursing, University of California, San Francisco, CA, United States

**Keywords:** telemedicine, access, health equity, scoping review, healthcare utilization

## Abstract

**Introduction:**

The COVID-19 pandemic led to a dramatic increase in telemedicine use for direct patient care. Inequities in device/internet access can limit the extent to which patients can engage with telemedicine care and exacerbate health disparities. In this review, we examined existing literature on interventions designed to improve patient telemedicine access by providing digital devices including tablets, smartphones, and computers and/or internet connectivity.

**Methods:**

In this systematic scoping review, we searched four databases for peer-reviewed studies published 1/1/2000–10/19/2021 that described healthcare interventions that provided patients with devices and/or internet connectivity and reported outcomes related to telemedicine access and/or usage. Data extraction elements included: study population, setting, intervention design, details on device/connectivity provision, and outcomes evaluated.

**Results:**

Twelve articles reflecting seven unique interventions met inclusion criteria. Ten articles examined telemedicine utilization (83%) and reported improved patient show rates/utilization. Seven articles examined patient satisfaction with the interventions (58%) and reported positive experiences. Fewer articles examined health outcomes (17%; 2/12) though these also demonstrated positive results. Across included studies, study quality was low. There were no controlled trials, and the most rigorously designed studies (*n* = 4) involved pre/post-intervention assessments.

**Discussion:**

Findings from this review indicate that providing material technology supports to patients can facilitate telemedicine access, is acceptable to patients and clinicians, and can contribute to improved health outcomes. The low number and quality of existing studies limits the strength of this evidence. Future research should explore interventions that can increase equitable access to telemedicine services.

**Systematic Review Registration:**

https://www.crd.york.ac.uk/prospero/display_record.php?RecordID=183442, identifier, PROSPERO: CRD42020183442.

## 1 Introduction

Use of telemedicine increased exponentially in response to the COVID-19 pandemic (1). In comparison to in-person care, synchronous virtual services (e.g., phone, live video-conferencing) has been shown to lead to comparable health outcomes (2–4). Furthermore, studies suggest that increasing access to virtual medicine can improve health outcomes for patients who are unable to access in-person care (5–9). However, marked inequities in access to the devices and data needed to utilize telemedicine (one aspect of the “digital divide”) means not all patient populations can access telemedicine services (10). The digital divide's impact on virtual care access was especially concerning at the height of the COVID-19 public health emergency, when in-person care was suddenly and markedly restricted due to disease transmission concerns (11, 12). The digital divide continues to threaten health equity given the volume of care delivered by telemedicine even as the pandemic abates (13). This can be especially relevant for patients living in rural settings, patients with transportation barriers, or patients with other socioeconomic barriers to attending in-person visits.

Achieving telemedicine equity requires equitable access to devices, internet/phone services (ideally broadband for video connectivity), technology skills/digital literacy, and technology supports (including technical assistance, training). Differences in access to these elements across demographics and geographic areas constitutes the digital divide, which is observed across age, race/ethnicity, and socioeconomic groups. Approximately 21 million people in the United States lack broadband internet access (14)—the ideal modality of internet connection for telemedicine use, given that cellular data plans often do not provide as reliable or strong an internet connection (especially on limited data plans). Poor digital literacy is a significant barrier to telemedicine use in older patients and patients with lower educational attainment (15). Patients who are older than 65, Black, Hispanic, Spanish language-preferring, or living in a low-income household are less likely than other patients to use video visits for health care (16, 17). Yet patients facing challenges related to digital literacy are nonetheless interested and willing to use telemedicine (9). Disparities in connectivity and literacy across patient populations raise important questions about the potential impact of telemedicine on health equity. Telemedicine has the potential to improve access to care for patients with barriers to in-person services, but if those same patients cannot access telemedicine services, telemedicine may instead worsen inequities in healthcare access and health itself.

Devices and connectivity are foundational to any telemedicine access. In this systematic scoping review, our objective was to identify and evaluate studies that have assessed the impact of interventions to provide patients with devices and/or connectivity on telemedicine access and/or other health and healthcare outcomes. We wanted to know how many studies have attempted to provide devices and/or connectivity to patients, and what outcomes have been evaluated.

## 2 Methods

### 2.1 Search terms and data sources

In collaboration with an experienced medical librarian, three study team members (J.B., E.H.D., N.V.S.) developed and refined an initial search to identify articles published in the peer-reviewed literature from 1/1/2000 to 7/20/2020 that described health care-based interventions to reduce disparities in telemedicine access. The initial search used a four-concept strategy including terms for: (1) telemedicine, (2) access, (3) disparities, and (4) interventions. Telemedicine was defined as real-time video or telephone communication between an outpatient clinician and patient (synchronous services); we excluded asynchronous services like electronic health record (EHR) messaging between a clinician and patient (which fall under telehealth services more broadly) (18). Access terms focused on internet, device, and/or medical record access. Disparities-related terms focused on health disparities and included a range of socioeconomic and digital literacy terms, including “disparities,” “poverty,” “computer literacy,” and “tech.” Intervention terms were used to identify articles that included an intervention. See Supplementary Appendix S1 for additional search information. We subsequently updated the search to capture literature published between 7/20/2020 and 10/19/2021 that focused on interventions to provide devices and/or internet for telemedicine services and described a related outcome from the intervention. Based on our experience with our initial search, and in consultation with an experienced medical librarian, we refined our original search terms to focus on (1) outcomes, (2) access, and (3) telemedicine. Outcomes terms focused on the most commonly reported outcomes from the original search, including effectiveness, care access, and intervention acceptability. Access terms focused on device access. Telemedicine terms remained similar to the original search strategy. See Supplementary Appendix S1.

We conducted our searches in four databases: PubMed (RRID:SCR_004846), EMBASE (RRID:SCR_001650), Web of Science (RRID:SCR_022706), and CINAHL (RRID:SCR_022707). Grey literature available through Web of Science and EMBASE was reviewed for inclusion. The search was adapted for each included database. We used Web of Science to perform a cited reference search and reviewed bibliographies for articles that met inclusion criteria to identify additional articles for screening.

### 2.2 Inclusion and exclusion criteria

To be included in this review, articles had to describe health care-based interventions to provide devices and/or broadband internet for synchronous remote telemedicine services. Interventions could address telehealth access more broadly, but had to include synchronous remote care, either by phone or video. We excluded studies where a device was provided solely to facilitate testing of another intervention, like a mobile application or remote monitoring device. We excluded studies conducted outside of the United States (U.S.), given unique features of the U.S. healthcare system around financing and reimbursement that may contribute to telemedicine access and the feasibility of health care-based interventions. Article full texts had to be available in English.

### 2.3 Data screening

After removing duplicates, search results were uploaded to a group library in Zotero 5.0.96.2 reference manager. Title and abstract screening were completed by two independent reviewers (J.B. and N.V.S.) in Zotero. Articles were excluded by title/abstract if they did not mention device and/or connectivity. If title/abstracts were too vague to determine provision of device(s) or connectivity, the article moved onto full-text review. Full-text screening was completed in an extraction spreadsheet by two independent reviewers (J.B. and N.V.S.). Following full-text screening, every study recommended by either reviewer was reviewed by an additional study author (E.H.D.). When differences of opinion between reviewers arose, they were resolved in discussion at both the title/abstract and full text levels. Cited reference searches of the final set of articles were performed in Web of Science. Bibliography reviews were conducted for the final set of articles.

### 2.4 Data extraction

Extraction tables were developed to record consistent data from each full text reviewed article, and included: study design, setting, patient population (including attention to race/ethnicity and language), intervention (e.g., provision of device and/or internet), and outcomes evaluated (e.g., health, healthcare utilization, cost, satisfaction). We followed PRISMA Extension for Scoping Reviews (PRISMA-ScR) reporting guidelines (Appendix 2) (20). The review was registered with the International Prospective Register of Systematic Reviews (CRD42020183442).

## 3 Results

**Figure 1:**
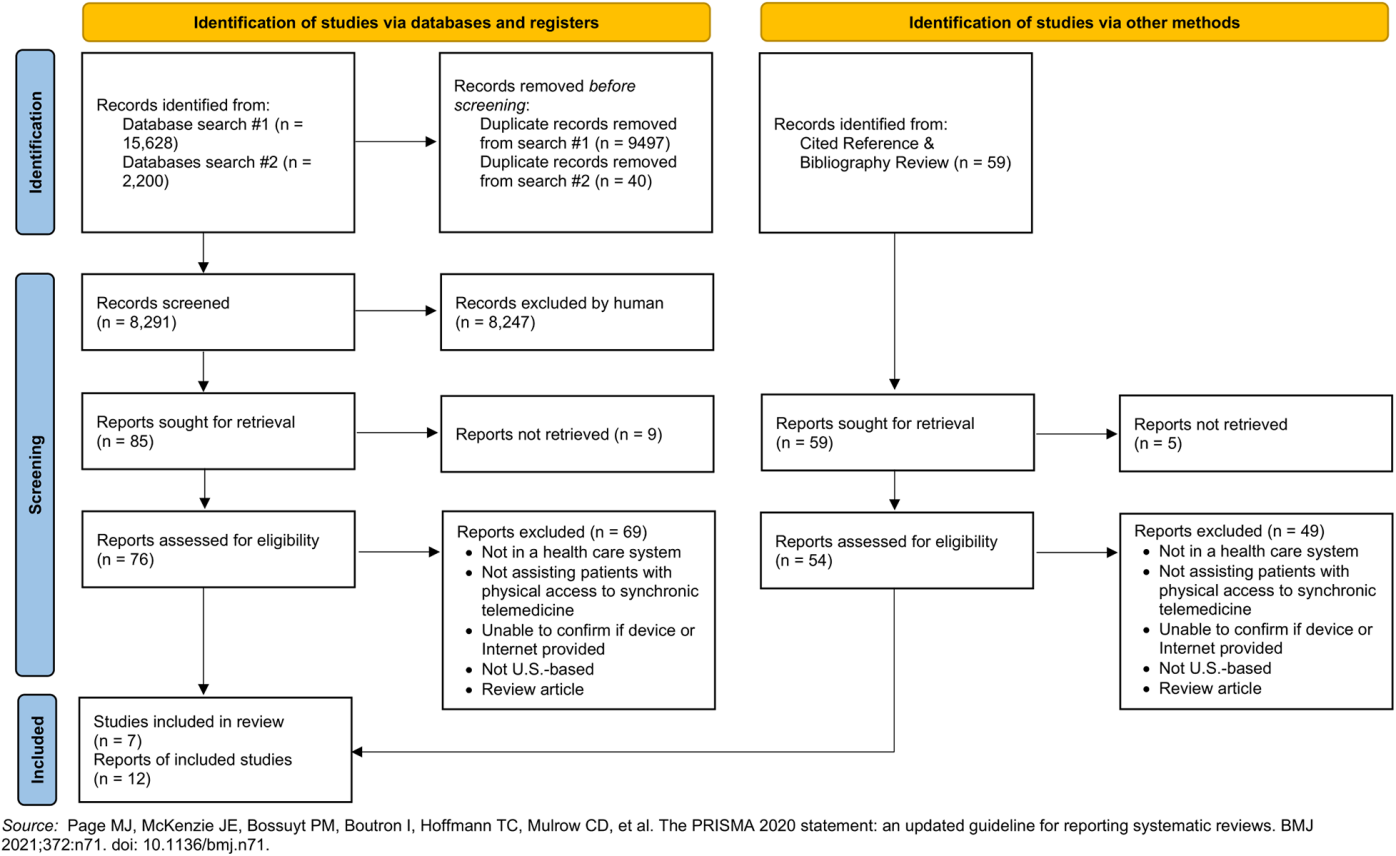
PRISMA 2020 flow diagram for systematic scoping review.

**Figure 2:**
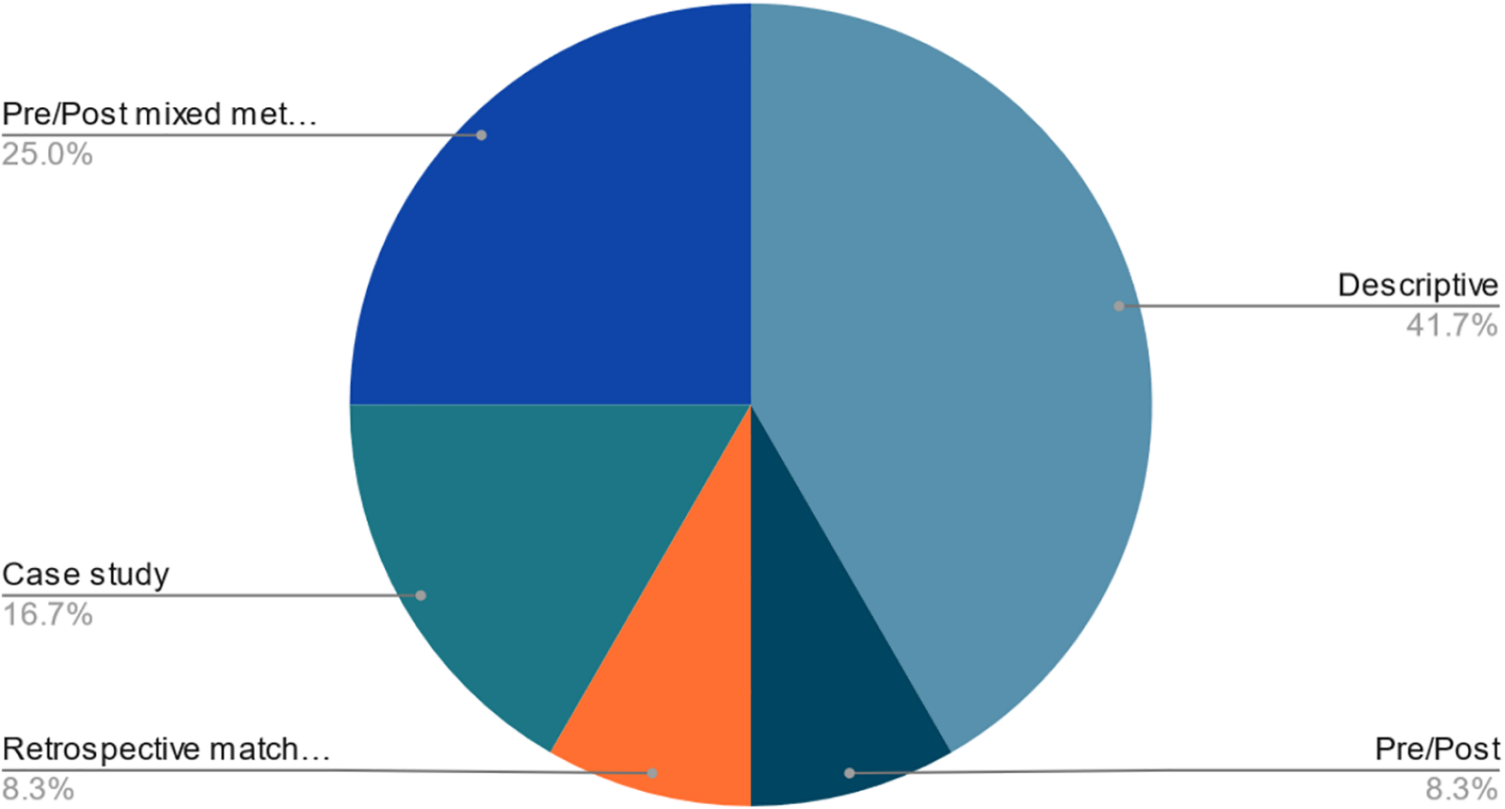
Distribution of study designs for the 12 included articles. The greatest percentage of articles (41.7%) use a descriptive study design. 25.0% of articles used a pre/post-intervention design with mixed methods, and 16.7% of articles were case studies. 8.3% of articles used solely a pre/post-intervention design, and 8.3% of articles used a retrospective matched cohort study design.

The initial search yielded 6,131 unique articles; 44 underwent full-text review. The second search yielded 2,160 unique articles; 41 underwent full-text review. By cited reference search and bibliography review of articles from the database searches that met inclusion criteria, an additional 59 articles were reviewed. In total, 130 articles underwent full-text review. Twelve unique articles met inclusion criteria across the searches (see Figure 1). Five articles (41.7%) were from studies using descriptive designs. Three articles (25.0%) used pre/post-intervention designs with mixed methods. Two articles (16.7%) were case studies, and one article (8.3%) used solely a pre/post-intervention design. One article (8.3%) used a retrospective matched cohort study design (Table 1, Figure 2). Some studies provided services to specific patient populations, e.g., 8/12 (67%) articles described interventions specific to veterans.

**Table 1:** Summary of included articles. Data from all articles meeting inclusion criteria are detailed here, including author, year of publication, study design, study setting, study population, the type of technology provided by the study, and key outcomes reported in each article.

Author, year	Study design	Study setting	Study population^a^	Technology provided	Outcome
Jacobs (20)	Retrospective matched cohort	U.S.A., nationwide	Veterans (*n* = 5,074); Race/Ethnicity: 76.5% non-Hispanic White, 12.8% non-Hispanic Black, 5.6% Hispanic, 4.0% Other; Language criteria not included	Tablet	Tablet recipients experienced an increase in psychotherapy visits (from 0.80 to 2.42 sessions) and medication management visits (from 1.60 to 1.80 sessions); the control group experienced a decline in these outcomes (0.63–0.30 and 1.21–0.36 respectively); 20.60% of tablet recipients met the VHA's continuity-of-care measure (patients with mental illness receiving three or more psychotherapy visits in a 6-week period) compared to 2.55% of patients who did not receive tablets; tablet recipients had a 20.24% lower rate of missed appointments than patients who did not receive tablets
Zulman (21)	Descriptive	U.S.A., nationwide	Veterans (*n* = 6,745); Race/Ethnicity: 72% non-Hispanic White, 11% non-Hispanic Black, 5% Hispanic, 5% Other, 5% unknown; language criteria not included	Tablet	81% of recipients used the tablets, primarily for mental health care; 86% of patients and 82% of providers responded well to the initiative
Slightam (22)	Pre/post mixed methods	U.S.A., nationwide	Veterans (*n* = 744); Race: 80.4% White, 12.1% Black or African American, 2.4% American Indian/Native Hawaiian/Other, 0.5% Asian, 4.6% unknown; Ethnicity: 4.2% Hispanic or Latino, 92.6% Non-Hispanic/Latino, 3.2% unknown or declined; language criteria not included	Tablet	Barriers centered around transportation and health-related challenges, outside commitments, and feeling uncomfortable at the VA; satisfaction with the tablet program was high by the majority of measures, including tablet arrival time, ease of use, and security; patients involved in the initiative had an average of approximately one tablet encounter per month over a 6-month period; 32.1% of tablet recipients preferred care via a tablet to in-person care, and 35.7% care via a tablet “about the same” as in-person care (patients in these two groups were more likely to feel uncomfortable being on site at a VHA facility, report a collaborative relationship with their provider, have a substance use disorder, or live in an area with better broadband coverage); ∼45% of patients reported experiencing technological difficulties during video calls; >60% of patients felt they had enough technical support and trusted their tablets to work
Garvin (23)	Descriptive	U.S.A., nationwide	Unhoused veterans (*n* = 1,470); Race/Ethnicity: 51.4% non-Hispanic White, 37.1% non-Hispanic Black, 4.8% Hispanic, 6.6% Other; Language criteria not included	Tablet	15.9% more unhoused veterans used video visits for mental health than housed veterans; 4.4% fewer unhoused veterans used video visits for primary care than housed veterans; 11.6% fewer unhoused veterans used video visits for specialty or other care than housed veterans; Compared to unhoused non-users, there were 11.8% more unhoused tablet users in the 18–44 y/o age range, 13.7% more unhoused tablet users living in a rural location, and 12.1% more unhoused tablet users who require an hour or longer drive to the VA
Jacobs (24)	Pre/post mixed methods	U.S.A., nationwide	Veterans (*n* = 764); Race distribution not reported; Ethnicity distribution not reported; Language criteria not included	Tablet	92% of respondents reported tablets saved them money or time; 89% reported saving money, and 71% reported saving time; patients were more likely to report money savings if they lived further from the VA or experienced travel barriers; patients were more likely to report time savings if they were employed, reported more technology experience, were <45 years of age, or >65 years of age
Brearly (25)	Pre/post mixed methods	North Carolina, U.S.A.	Veterans (*n* = 20); Race: 70% White, 25% Black, 5% American Indian; Ethnicity distribution not reported; language criteria not included	Tablet	Mean reduction in post-discharge wait times of 18.6 days; Reductions in average scores on GAD-7 (−4 points), PHQ-9 (−4.5 points), DSM-5 PTSD checklist (−5 points), DSM level 2 anxiety (−1.1 points) and depression (−8.25 points) post-intervention; barriers to on-site care that included distance from the clinic (29%), lack of transportation (24%), the extent of the onsite clinic wait time (24%), anxiety from driving or riding in a car (18%), and difficulty taking time off of work (6%)
Sorocco (26)	Case study	Oklahoma, U.S.A.	Veterans (*n* = 6); Race distribution not reported; Ethnicity distribution not reported language criteria not included	Video capabilities	Case studies suggested improvements in physical strength, social functioning, and compliance with treatment plans; telemental health was successful in supporting treatment goals of the veteran
Doolittle (27)	Descriptive	Kansas and Missouri, U.S.A.	Hospice patients (*n* = 109); Race distribution not reported; Ethnicity distribution not reported Language criteria not included	Videophone	In traditional care, there was a total operational cost of $231,613 and a cost per patient visit of $126 in 1997; For telehospice, there was a total operational cost of $3,165 and a cost per patient visit of $29; In telehospice care, an additional 599 patient-days of care took place over three months compared to traditional care
Utley (28)	Case study	Los Angeles, California, U.S.A.	Patients 65 years old or older (*n* = 9); Race distribution not reported; language criteria not included	Smartphone	3 of 9 participants successfully contacted healthcare providers; patient concerns included WiFi affordability, invasion of privacy, recording and storing of video encounters, and preference for telephone appointments
Shem (29)	Descriptive	San Jose, California, U.S.A.	Patients with spinal cord injury (*n* = 10); Race distribution not reported; Ethnicity: 60% non-Hispanic White, 40% Hispanic; English-speaking only	Tablet	Half of the ten participants did not use telemedicine; 16 telemedicine visits occurred over 6 months; 10 ER visits and 4 hospitalizations occurred; 80% of ER visits were for participants who did not use telemedicine; 100% of hospitalizations were for participants who did not use telemedicine; 100% of patients reported positive experiences and wanted to continue the program; No significant differences in quality of life measures; 75.0% of respondents either strongly agreed or agreed that they were satisfied with the video and audio quality
Sechrist (30)	Descriptive	San Jose, California, U.S.A.	Patients with spinal cord injury (*n* = 62); Race/ethnicity: 55% non-Hispanic White, 23% Hispanic, 12% non-Hispanic Asian, 5% non-Hispanic African American, 5% Other; English-speaking only	Tablet	161 telemedicine visits occurred over 6 months; 57/62 participants completed the program; 45/57 participants completed the patient satisfaction survey; 100% of patients completing the survey would recommend the program; 88.0% of patients felt cared for through telemedicine; 55.56% of patients preferred telemedicine; 35.56% preferred in-person visits; 82.3% of respondents either strongly agreed or agreed that they were satisfied with the video and audio quality
Whealin (31)	Pre/post	Rural Pacific Islands, U.S.A.	Veterans (*n* = 47); Race: 27.6% Native Hawaiian/Pacific Islander, 25.6% White, 21.3% Asian American, 19.1% mixed, 6.4% Black; Ethnicity distribution not reported; English-speaking only	Tablet	Most veterans rated their satisfaction and the usability of the program high; 96.5% of patients agreed or strongly agreed that they would recommend the program to other veterans; 72.4% of patients preferred home telemental health services over in-person care; 7% patients had concerns about safety over HTMH; 11% of patients agreed that technical disruptions affected their overall satisfaction

^a^ Study population details as reported in articles. Data includes study population race, ethnicity, language. Different articles included different race and/or ethnicity categories and not all studies reported on race, ethnicity, and/or language.

The majority of interventions (8/12; 67%) took place in primary care settings; 3/12 articles (25%) reported on telemedicine services for outpatient mental health care and 1/12 (8%) for home hospice care. Eight of 12 articles (67%) were from studies from the Veterans Health Administration (VHA). Nine articles (75%) provided tablets, two articles (17%) provided smartphones/videophones, and one article (8%) provided computers. Eight articles (67%) offered technology support services in addition to devices. None of the studies provided only internet connectivity, though seven articles (58%) provided internet or data plans in addition to devices to facilitate device usage. No studies compared device provision with and without internet connectivity provision. The 12 articles examined a range of outcomes, including: (1) healthcare utilization, (2) patient experiences and perception of intervention acceptability, and (3) health impacts. Articles could examine outcomes from more than one of these categories. Results are summarized below by outcome.

### 3.1 Telemedicine visit access/use

Ten of 12 articles (83%) reported on healthcare utilization. Four (40%) of these articles were published on a nationwide intervention in the VHA that provided tablets and data plans to patients reporting barriers to accessing in-person health care. Two articles from this intervention used pre/post-intervention methods. One of the VHA articles that used a pre/post-intervention design focused on the effect of tablet provision on patients with mental health diagnoses. This article reported increased psychotherapy and medication management visits, improved continuity of care, and decreased missed appointments post-receipt of a tablet (20). Three other articles from the same VHA intervention (21–23) found similar results, including within other subpopulations of veterans, i.e., not focused on patients with mental health diagnoses (Table 1).

A separate pre/post-intervention study at the VHA in North Carolina provided patients with tablets to increase attendance at mental health medication management appointments. This study found that the time from discharge to first medication management appointment was decreased by an average of 18.6 days when compared to in-person appointments (25). Another study provided VHA patients in rural Oklahoma with telemedicine video and remote health monitoring equipment, which resulted in a substantial increase in the frequency of home-based primary care encounters compared to pre-intervention (26).

Four articles included information on the number of visits conducted by telemedicine after device provision (27–30). These articles did not include comparison groups, but they demonstrated the feasibility of telemedicine services after device provision. One study in patients with spinal cord injuries did report that ED visits and hospitalizations were lower among patients who used telemedicine services (29).

### 3.2 Patient satisfaction with telemedicine vs. in-person visits

Seven of 12 articles (58%) examined patient perceptions of the implementation of telemedicine, including assistance with devices and/or internet. These articles did not distinguish between satisfaction with receiving devices/internet and satisfaction with the associated interventions. In one article from the nationwide VHA study providing tablets to patients with barriers to in-person access, the majority of patients found the initiative to be an acceptable method of receiving care (21). Another article from the same intervention focused on patient perceptions of the telemedicine initiative and reported high patient satisfaction; approximately two-thirds of participants either preferred care via tablet or rated care via tablet as “about the same” as in-person care (22).

In a pre/post-intervention study that provided VHA patients in the rural Pacific Islands with tablets to participate in remote PTSD therapy, most of the veterans reported positive experiences with program usability and preferred home telemental health services to traveling to the VHA hospital (31). Out of 28 patients, few had concerns regarding safety (7%) or technical disruptions (11%) (the article did not provide details on safety concerns) (31). In the North Carolina-based VHA study, participants cited various barriers to on-site care that made the telemedicine program desirable (25). The most common barriers reported were distance from the clinic, lack of transportation, and onsite clinic wait times (25).

The study conducted with spinal cord injury patients formally assessed patient perceptions using a patient satisfaction survey at the end of the 6-month study period (29, 30). One article from the study found that all 10 participants reported positive experiences with the telemedicine services and wanted to continue with the program (29). Additionally, patients appreciated the ability to use the provided tablets for activities separate from telemedicine, such as general internet access, videos, and music (29). In the second article, 57/62 participants completed the program, and all 45/57 participants who completed the patient satisfaction survey reported they would recommend the program to others (30). Over 50% (*n* = 25/45, 56%) preferred telemedicine to in-person, whereas 36% (*n* = 16/45) preferred in-person visits (30).

In a descriptive study providing older patients with smartphones, patients reported concerns about the cost of WiFi, that telemedicine was an invasion of privacy (e.g., some patients did not want their providers to see their homes), and that video encounters would be recorded and stored (although they were not) (28). Additionally, some patients preferred telephone appointments to video appointments using the smartphone; no information was provided on why (28).

#### 3.2.1 Patient-reported benefits of telemedicine visits

One of the 12 articles (8%) reported on additional patient-reported benefits of telemedicine visits. This article from the nationwide VHA tablet study found that patient participants' self-reported money and time savings related to telemedicine use (24). This included savings in transportation, gas, lodging, and food. Eighty-nine percent of patients reported saving money, and 71% reported saving time (24).

#### 3.2.2 Patient-reported technical barriers to telemedicine visits

Four of the 12 articles (33%) included data on the technological performance of the devices provided to patients. One of the articles from the nationwide VHA study used patient-reported experiences to gauge technology performance. Although many patients reported experiencing technological difficulties during video calls, over 60% of 590 patients felt they had enough technical support and trusted their tablets to work (22). In the Pacific Islands-based VHA study, 61% of 28 participants either strongly disagreed or disagreed that technical disruptions impacted their overall satisfaction with the program; 11% strongly agreed or agreed (31).

Both studies conducted with patients with spinal cord injury assessed technology performance through the program satisfaction survey using a question on video and audio quality. Seventy-five percent of respondents in the 10-participant study were satisfied with video and audio quality (29). In the 62-participant study, 82% of respondents were satisfied with video and audio quality (30).

### 3.3 Health outcomes

Only two of 12 articles (17%) evaluated health outcomes of their interventions. Neither of the studies included comparison groups, and no studies evaluated chronic disease management. In a VHA-based intervention in rural Oklahoma, improvements were observed in patient physical strength and social functioning over the 6-month study period, measured by virtual occupational therapy evaluation and self-report (26). The North Carolina-based VHA study compared pre- and post-intervention psychological outcomes and noted reductions in scores for the Generalized Anxiety Disorder (GAD)-7, Patient Health Questionnaire (PHQ)-9, and Post-Traumatic Stress Disorder (PTSD) Checklist for Diagnostic and Statistical Manual of Mental Disorders (DSM-5), as well as reductions in scores in DSM level 2 anxiety and depression (25).

## 4 Discussion

Although ensuring adequate device access and internet connectivity is foundational for equitable telemedicine adoption, we found only 12 studies focused on providing devices and/or internet connectivity to improve telemedicine access. Our findings provide early evidence that increasing patient access to digital devices and/or broadband internet can increase visit attendance but evidence for improvement in health and treatment outcomes remains sparse particularly in the absence of high-quality studies with a contemporaneous control group. There are also many additional potential benefits to patients experiencing and/or at risk of health inequities, including lower out-of-pocket costs for travel to clinic visits, which may help improve adherence to scheduled clinic visits needed for effective chronic disease management.

Few studies evaluated the impact of telemedicine access on health outcomes. This is most likely a result of the early feasibility focus of this body of literature, first addressing the acceptability and effects of interventions on healthcare utilization, the latter of which was occasionally discussed as a proxy for physical health. Although it is encouraging that technology provision appears to be feasible and beneficial across multiple care settings, including mental health, hospice, and primary care, it is also important to acknowledge that there is less evidence on how devices/connectivity can impact health outcomes and related, health equity. Device and internet access are necessary for the effective utilization of telemedicine, yet many studies overlook access (i.e., do not acknowledge or assess device access or connectivity), or conflate access with other important related but critically distinct aspects of technology uptake (such as patient technology skills). Future studies will need to examine patients' device access and connectivity when evaluating the impact of telemedicine on health outcomes to better assess how different components of telemedicine access impact health equity and inform interventions to advance health equity.

As material supports for virtual care evolve, achieving equity will require careful attention to program design and evaluation. The studies included in this review reflected little diversity (e.g., by race, gender, language), when relevant data were provided. Future studies should strive to increase participant diversity to better understand the impact of material technology supports in different patient populations. The studies in this review either did not report on participants' preferred language or required English literacy as part of their inclusion criteria. Yet prior work has shown that preferred language may have a considerable impact on telemedicine experiences and preferences, with Spanish-preferring patients encountering more technical challenges and having greater preference for in-person visits (32). Addressing these weaknesses in the current literature both could help to strategically identify subgroups most adversely affected by resource inequities and enable healthcare systems to better target future interventions to improve telemedicine and health equity (33).

Our review should be interpreted in light of several limitations. Most importantly, we found only 12 articles meeting inclusion criteria, which, combined with variable study designs and small sample sizes, limits the generalizability of findings. A majority of articles were conducted within the VHA; this may limit the generalizability of findings to non-VHA settings. No studies had a control group, and only four had pre/post-intervention designs (20, 22, 25, 31). Many studies that provided devices to patients were excluded from this review because their primary focus was to test an application, not for synchronous telemedicine encounters. No studies in this review identified implementation approaches to improve telemedicine uptake without increasing staff workload, despite the common recognition that time and lack of training are barriers to telemedicine adoption (21, 25, 26). All of these factors limit the evidence base for device/connectivity provision. It is all the more important that more studies implement and rigorously analyze interventions to improve telemedicine access for populations with low rates of broadband access or digital device ownership. A greater number of higher quality, comparative effectiveness studies is necessary to determine the most effective ways to close the digital divide and prevent the widening of health disparities. Given that telemedicine is likely to continue to be a common modality for delivering health care and holds promise for decreasing barriers to traditional care, e.g., for patients facing transportation, childcare, and/or employment barriers to in person care, it may be useful to reassess the literature in this space as more work emerges about the impact of device/connectivity provision. We acknowledge we included literature through October 2021—relevant articles that could have added to the evidence may have been published since, but resource limitations prevented further review updates. Given the evolving landscape of telemedicine, we recommend repeating this review in the next 5–10 years.

Second, distinguishing the impact of device provision from the impact of technical support/training in closing the digital divide is difficult. Many of the interventions captured in this review provided technology-related support services alongside the material supports that were the focus of this review, muddling the association between device/data provision alone and healthcare access outcomes. Separating device provision from technical support may be artificial as the two often coexist (and likely for good reason). Similarly, participants' perceptions of the interventions were likely influenced by the quality of technology support provided. This support sometimes entailed addressing patients' lack of digital literacy. This creates further challenges in distinguishing which of the elements of the digital divide that were addressed contributed to the outcomes of interventions and how. Future research should report on both the device/connectivity access as well as training/skills and technical support provided to patients to improve our understanding of key intervention functions to improve telemedicine access. While the focus of this review was interventions providing technology directly to patients, this is not the only way to alleviate access barriers. Our review did not include studies that used a secondary public location, such as a library, to facilitate virtual care. This could be an alternative method to increase device/data access, often with the benefit of on-site technical support. One study examining this approach showed improvements in telemedicine readiness. The greatest barrier identified was establishing a private space within the library (34).

In conclusion, few studies have examined the impacts of providing material technology supports to improve telemedicine access and healthcare outcomes. Despite the low number and primarily descriptive design of studies included in this scoping review, the early research suggests that healthcare-based interventions to provide patients with telemedicine technology can positively impact care access, patient experience, and health, which may all help to improve health equity in the long-term. Future research should more rigorously explore the role of material technology supports in closing the digital divide and as a strategy for improving health equity.

## Data Availability

The original contributions presented in the study are included in the article/Supplementary Material, further inquiries can be directed to the corresponding author.
