# Supplementary material for: Provision of digital devices and internet connectivity to improve synchronous telemedicine access in the U.S.: a systematic scoping review
Source: Front Digit Health. 2024 Jul 29;6:1408170. doi: 10.3389/fdgth.2024.1408170 (PMC11317370; doi:10.3389/fdgth.2024.1408170)
Supplement: Supplementary file 1 [file Datasheet1.pdf]

Appendix 1

Search 1:

(((((“telemedicine”[Mesh] OR "telemedicine"[tiab] OR “telehealth”[tiab] OR “mhealth”[tiab] OR “telecare”[tiab] OR “videoconsul*”[tiab] OR “ehealth”[tiab] OR "patient portal" [tiab])) AND ((“Internet Access”[Mesh] OR “Internet Access”[tiab] OR "broadband" [tiab] OR “Patient Access to Records”[Mesh] OR “Health Care Quality, Access, and Evaluation”[Mesh] OR “access”[tiab] OR “use”[tiab] OR "digital divide"[tiab] OR tablet[tiab]))) AND ((“Healthcare Disparities”[Mesh] OR “Health Status Disparities”[Mesh] OR “Minority health”[Mesh] OR “Race Factors”[Mesh] OR “disparities”[tiab] OR "language"[tiab] OR "health literacy"[tiab] OR "rural"[tiab] OR "urban"[tiab] OR "race"[tiab] OR "poverty"[tiab] OR "socioeconomic"[tiab] OR "computer literacy"[tiab] OR "tech"[tiab] OR "literate" OR "literacy" OR “socioeconomic factors”[mh] OR barrier*[tiab]))) AND ((interven*[tiab] OR addressing[tiab] OR provision[tiab] OR linking[tiab] OR incorporat*[tiab] OR connecting[tiab] OR program development[tiab] OR implement*[tiab] OR partner*[tiab] OR distribut*[tiab] OR adopt*[tiab])))

Search 2:

(effectiveness OR evaluation OR costs OR perception OR access OR barriers) AND (tablet OR tablets OR equipment) AND (telehealth[ti] OR telerehab[ti] OR telemedicine[ti] OR telemental[ti])
